# Perivascular epithelioid cell tumor of the uterus and pelvic cavity

**DOI:** 10.3389/fonc.2024.1449936

**Published:** 2024-10-30

**Authors:** Xiuzhang Yu, Ruiqi Duan, Bowen Yang, Liyan Huang, Minmin Hou, Mingrong Qie

**Affiliations:** ^1^ Department of Obstetrics and Gynecology, West China Second University Hospital, Sichuan University, Chengdu, China; ^2^ Key Laboratory of Birth Defects and Related Diseases of Women and Children (Sichuan University), Ministry of Education, Chengdu, China; ^3^ Department of Pathology, West China Second University Hospital, Sichuan University, Chengdu, China

**Keywords:** perivascular epithelioid cell tumor, female reproductive system, surgical intervention, invasiveness, pulmonary metastasis

## Abstract

**Background:**

Primary perivascular epithelioid cell tumors (PEComas) of the female reproductive tract have been primarily reported as case reports owing to their clinical rarity. Limited incidence rates and clinical case data hinder a comprehensive understanding of the risks and invasiveness of this disease. We discuss herein the diagnosis, treatment, and prognosis of this disease to enhance comprehension and therapeutic strategies.

**Methods:**

We conducted a clinical analysis of patients with PEComa treated at the Gynecology Department of The West China Second University Hospital of Sichuan University between May 2018 and January 2024. Diagnosis and treatment were evaluated based on pertinent literature.

**Results:**

Overall, eight patients (seven patients with tumors in the uterus and one patient with tumors in the pelvic cavity) were evaluated. One patient with PEComa of unknown malignant potential and two patients with malignant PEComa underwent hysterectomy and bilateral adnexectomy with or without adjuvant therapy and did not develop recurrence. Meanwhile, three patients who underwent lesion resection only exhibited radiological evidence of new lesions. Furthermore, postoperative imaging identified new pulmonary nodules in three patients.

**Conclusion:**

Although the current criteria are generally effective in assessing the tumor invasiveness of PEComa, emphasizing the significance of complete lesion resection remains crucial. Inadequate treatment significantly increases the risks of recurrence and metastasis. Additionally, the prevalence of pulmonary metastases may have been underestimated. Refining risk stratification to prevent overtreatment of low-grade malignancies or overlooking highly aggressive tumors is an important area for further study.

## 1 Introduction

Perivascular epithelioid cell tumor (PEComa) is an uncommon neoplasm encompassing angiomyolipoma, clear cell “sugar” tumor of the lung and extrapulmonary tissue, lymphangioleiomyomatosis, and nonspecific perivascular epithelioid cell tumor. These tumors are characterized by the World Health Organization as “mesenchymal neoplasms consisting of histologically and immunohistochemically distinct perivascular epithelioid cells.” Nonspecific PEComa refers to a PEComa affecting various sites such as the uterus, vulva, rectum, thigh, pancreas, abdominal wall serous membrane, or heart. It occurs most frequently in the uterus, mostly in the uterine body, but a few cases in the cervix have also been reported. Owing to its clinical rarity, primary uterine or pelvic PEComa is mainly reported in the literature as case reports. This study discussed the diagnosis and treatment of this condition based on pertinent literature to enhance comprehension and therapeutic strategies. We performed a clinical analysis of patients with PEComa (seven patients with tumors in the uterus and one patient with a tumor in the pelvic cavity) treated at the Gynecology Department of the Gynecology Department of The West China Second University Hospital of Sichuan University between May 2018 and January 2024.

## 2 Materials and methods

### 2.1 Patient selection and pathologic examination

Patients diagnosed with uterine or pelvic PEComa who were admitted and treated at The West China Second University Hospital of Sichuan University between May 2018 and January 2024 were evaluated. Patients with missing data on clinical diagnosis or treatment were excluded. The diagnosis of PEComa was pathologically confirmed.

This study was approved by the institutional review board of the West China Second University Hospital and was conducted according to the tenets of the Declaration of Helsinki. Informed consent was obtained from all the patients.

### 2.2 Data collection and follow-up

General data, clinical manifestations, auxiliary examinations, pathological features, treatment modalities, and patient prognoses were reviewed and analyzed. The patients were followed up via telephone interviews until January 6, 2024.

## 3 Results

### 3.1 Patient characteristics

A 33-year-old female with a cervical PEComa visited our hospital for outpatient treatment. She underwent hysterectomy, bilateral adnexectomy, greater omentum resection, and pelvic lymph node dissection at an external hospital. However, this patient was excluded from this study owing to insufficient clinical follow-up data. A total of eight patients aged between 33 and 52 years were included. One patient experienced amenorrhea due to tuberculosis, while three patients were menopausal. Except for the patient with amenorrhea, all patients had a history of fertility. Five patients previously underwent uterine surgery, including myomectomy, cesarean section, and total hysterectomy (TH). Two patients also had breast nodules, and one patient had a history of breast cancer surgery and chemoradiotherapy. Initial symptoms included postmenopausal vaginal bleeding in one patient, vaginal discharge in two patients, pelvic masses in one patient, and abnormal uterine bleeding characterized by increased menstruation and irregular vaginal bleeding in four patients. The patient characteristics are presented in **Table 1**.

**Table 1:** Clinical features of the patients.

Cases	Age	Meno-pausal	Fertility history	Medical and surgical history	Clinical presentation	Preoperative imaging examination
1	46	no	G4P1	cesarean section, cholecystectomy, breast nodules, hypothyroidism	increased menstruation	ultrasound: suspected uterine fibroids
2	48	no	G3P1	cesarean section; thyroid and breast nodules	irregular vaginal bleeding	CT: Mildly enhanced patchy soft tissue shadow in the uterus, with an unclear boundary between the soft tissue and the uterine wall muscle layer
3	51	yes	G2P2	TH for “multiple uterine fibroids”	the pelvic masses	ultrasound: suspected angioleiomyoma CT: multiple variable-sized nodules in both lungs
4	52	yes	G4P1	diabetes	vaginal discharge	ultrasound: rich blood flow signals detected in the mass
5	33	no	G0P0 (amenorrhea after contracting tuberculosis)	tuberculosis	vaginal discharge, uterine cavity occupancy	ultrasound: malignant lesions? CT: several small nodules in the right subpleural and interlobar regions
6	51	yes	G2P1	Myomectomy; surgery, chemoradiotherapy and targeted therapy for breast cancer	postmenopausal vaginal bleeding	MRI (a)
7	50	no	G3P2	Myomectomy	increased menstruation	ultrasound: submucosal myoma?
8	40	no	G4P1	ectopic pregnancy tubal resection surgery, cholecystectomy, diabetes	irregular vaginal bleeding	ultrasound: heterogeneous slightly strong echo

a. a nodular lesion measuring 1.5×0.7x1.6 cm in the anterior wall of the lower uterine segment. The T2WI signal exhibited high intensity, slightly lower than that of the endometrium, while the T1WI signal showed slightly higher intensity with restricted diffusion. The lesion involved more than half of the anterior wall muscle layer, demonstrating pronounced enhancement during early dynamic scans and mild enhancement in delayed scans.

### 3.2 Clinicopathological features

Most of the lesions were located in the uterine body (6/8), including the submucosa (n=4), intermuscular wall (n=1), subserous membrane (n=1), submucosa in the lower segment of the uterine body and the junction of the cervical body (n=1), and the pelvic cavity (n=1 patient who had post-TH). The maximum tumor diameter range was1.6–7 cm. Among the eight patients, five patients had PEComa, one patient had PEComa of uncertain malignant potential (UMP), and two patients had malignant PEComa. The intraoperative frozen pathological examination results are presented in **Table 2**. Only one patient was diagnosed with PEComa intraoperatively. This patient initially underwent hysteroscopic lesion resection, with pathology indicating PEComa. A subsequent surgery was performed involving laparoscopic total hysterectomy and bilateral adnexectomy. Intraoperative frozen section analysis also suggested PEComa. The final postoperative pathology revealed PEComa of UMP.

**Table 2:** Clinicopathological findings of the patients.

Cases	Tumor site	Diameter of lesion (cm)	Intraoperative frozen section pathological examination	Final pathological diagnosis	Other pathological diagnosis
1	subserosal	3	a mesenchymal tumor? an epithelioid smooth muscle tumor or a perivascular epithelioid cell tumor?	PEComa	leiomyoma
2	submucosal	Unknown	/	PEComa	adenomyosis of the uterus, simple endometrial hyperplasia, and complex endometrial hyperplasia
3	the pelvic cavity	7 + 4 (lobulated)	hyperplastic fibrovascular tissue with widespread infiltration of inflammatory cells	PEComa	/
4	intermuscular	1.8	/	PEComa	adenomyosis, leiomyoma
5	submucosal	5	extensive necrotic tissue with focal calcification and suspected granulomas, suggesting tuberculosis	PEComa	tuberculous changes (necrosis and granulomatous inflammation)
6	submucosal (in the lower segment of the uterine body and the junction of the cervical body)	1.6	PEComa (a)	PEComa of UMP	None
7	submucosal	5.3	the tumor predominantly exhibits epithelioid morphology, with visible mitotic figures (accurately counting the number of mitotic figures in frozen sections is difficult). Focal nuclear atypia is observed. Epithelioid leiomyoma is primarily considered, at least as epithelioid leiomyoma of uncertain malignant potential. Other tumors, such as PEComa and endometrial stromal tumors, also need to be excluded	malignant PEComa	multiple leiomyomas and adenomyosis
8	submucosal	4	/	malignant PEComa	leiomyoma

a. The patient initially underwent hysteroscopic lesion resection, with pathology indicating PEComa. A subsequent surgery was performed involving laparoscopic total hysterectomy and bilateral adnexectomy. Intraoperative frozen section analysis also suggested PEComa. The final postoperative pathology revealed PEComa of UMP.

**Figure 1:**
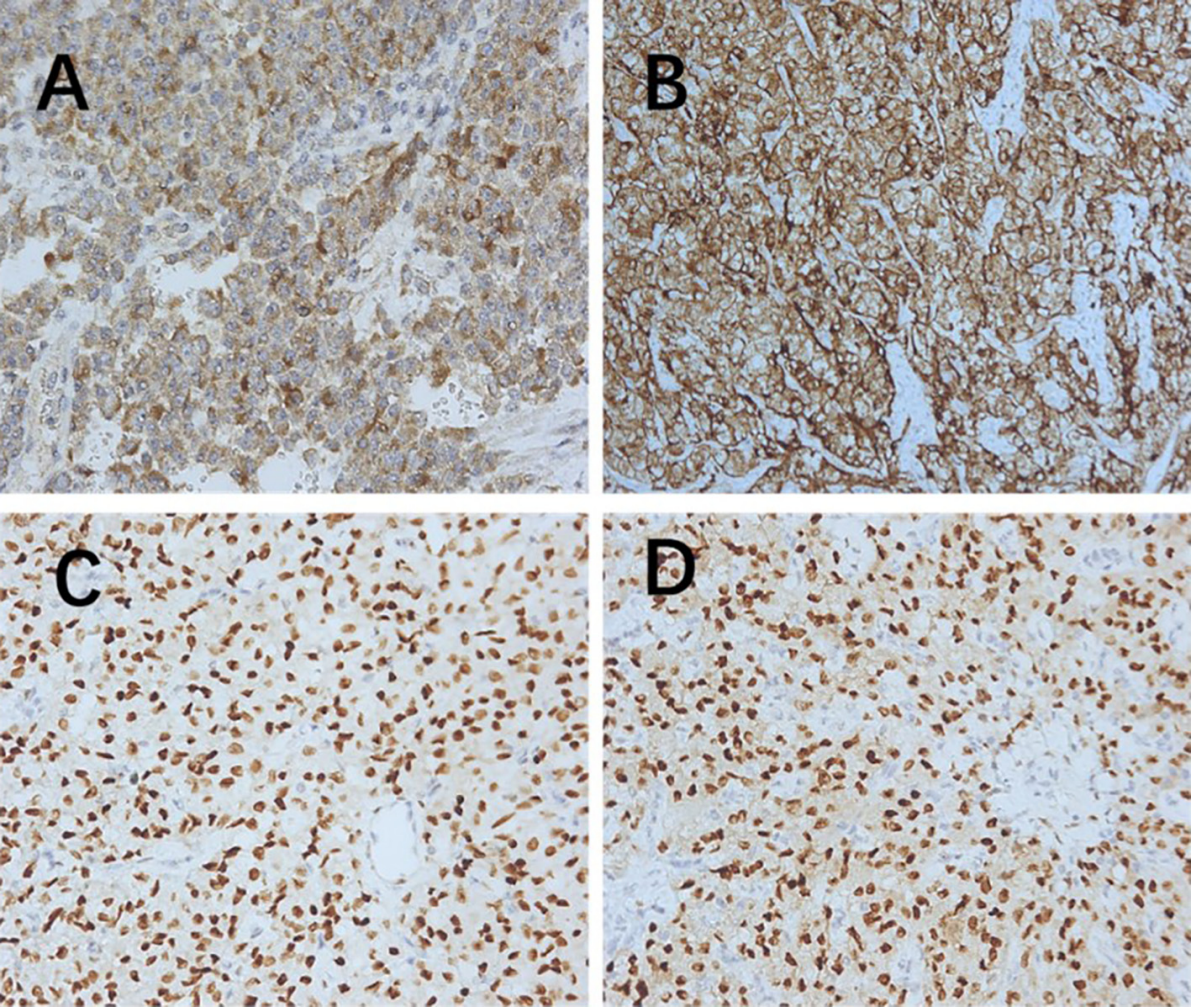
Immunohistochemistry (200× magnification). **(A)** Melanoma positive. **(B)** HMB45 positive. **(C)** Estrogen receptor positive. **(D)** Progesterone receptor positive. HMB45, human melanoma black 45.

**Figure 2:**
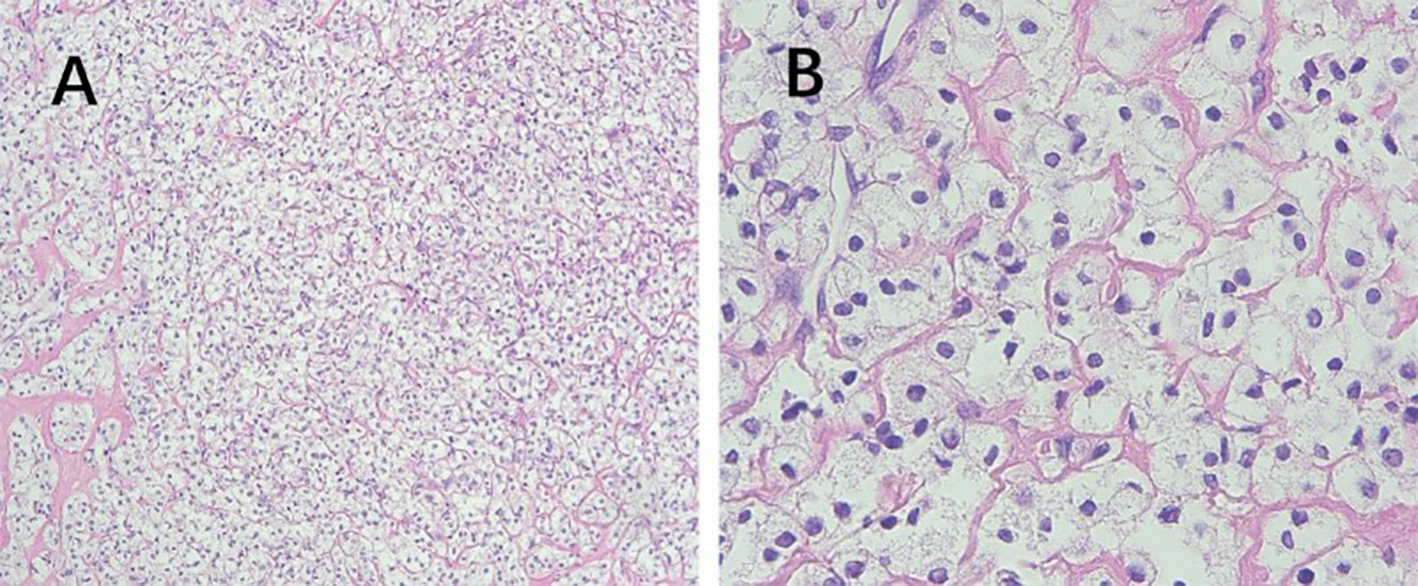
Pathological features of PEComa. **(A)** The epithelioid cells are arranged in nests or sheets, with clear cytoplasm, and delicate thin-walled small blood vessels are observed between the cell nests (100× magnification). **(B)** Absence of cell atypia, coagulative necrosis, or mitotic signs (400× magnification). PEComa, perivascular epithelioid cell tumor.

**Figure 3:**
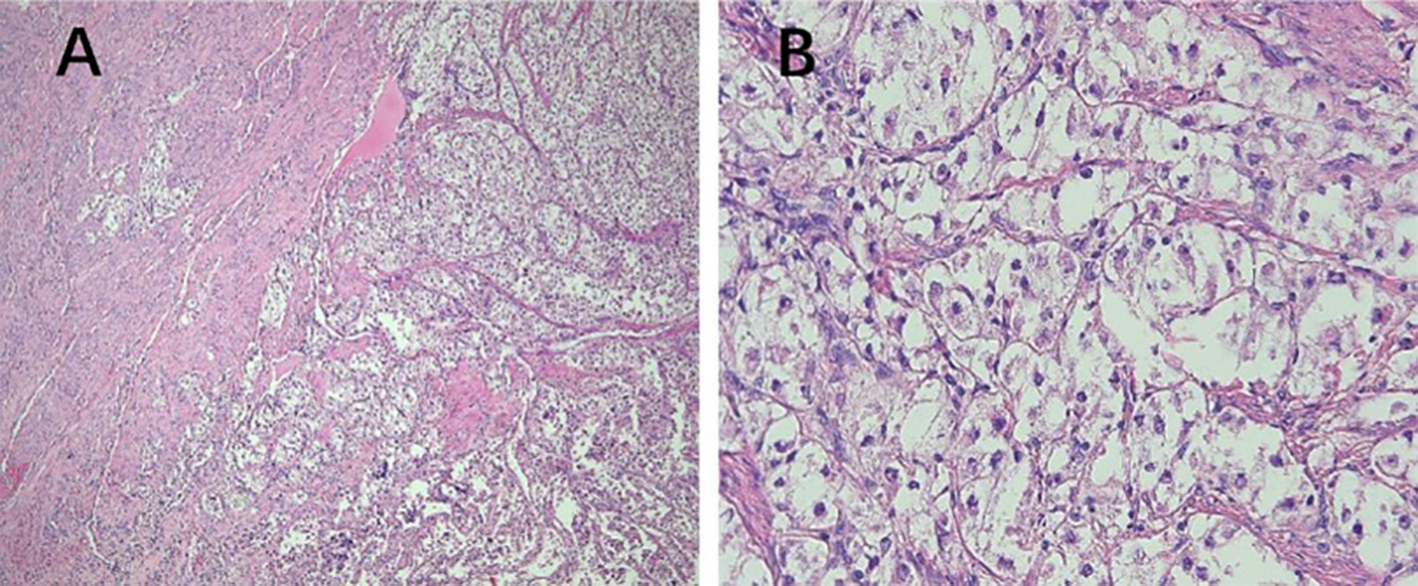
Pathological features of PEComa of UMP. **(A)** The tumor exhibits poorly defined borders, with no clear demarcation from the muscle layer (40× magnification). **(B)** The cells are arranged in nests, displaying clear cytoplasm with no evidence of cellular atypia (400× magnification). PEComa, perivascular epithelioid cell tumor; UMP, uncertain malignant potential.

**Figure 4:**
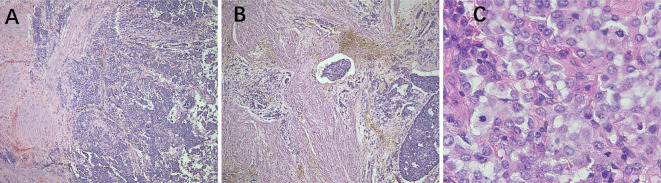
Pathological features of malignant PEComa. **(A)** The tumor exhibits unclear borders and infiltrates the muscle layer (40× magnification). **(B)** The tumor exhibits infiltrative growth within the muscle wall, with tumor emboli observed in the blood vessels (40× magnification). **(C)** Atypical epithelioid tumor cells and pathological nuclear division (400× magnification). PEComa, perivascular epithelioid cell tumor.

All patients underwent immunohistochemical analysis of melanocytes and myogenic differentiation markers. Genetic testing for transcription factor E3 (TFE3) conducted in one patient showed no significant findings (**Table 3**). Pathological images are shown in **Figures 1**–**4**.

**Table 3:** Immunohistochemical results of the patients.

Cases	HMB45	Melan-A	TFE3	S-100	Desmin	caldesmon	SMA	CD34	WT-1	Calponin	VIM	Calretinin	CyclinD1	ER	PR	ki67	Genes
1	–	+	/	–	+	–	/	blood vessel +	/	+	/	/	/	partial cells +	partial cells +	/	
2	/	+	/	–	+++	–	/	/	/	/	/	+	scattered cells+	+++	+++	4-5%	
3	++	+	/	/	+++	+++	++	–	/	+++	+++	/	/	++	++	2-3%	No meaningful TFE3 detected
4	focal +	+++	/	–	–	focal +	focal +	/	/	/	+++	/	/	–	–	3%	
5	+	focal +	/	–	/	–	–	/	/	/	partial cells +	/	/	–	+	20% (curettage), <10% (hysteroscopy)	
6	+++	focal +	/	–	–	–	–	/	/	/	/	/	+++	–	++	10%	
7	a few scattered cells +	+	+++	–	+++	focal +	weak +	–	+++	+++	/	focal +	scattered cells +	+++	+++	20%	
8	–	+	/	–	+	–	–	–	/	–	+	–	–	+	+	20%	

+ weak positive, ++ moderate positive, +++ strong positive, / not detected, - negative.

### 3.3 Diagnosis, treatment and prognosis

The follow-up period ranged from 9 months to 68 months, and all patients survived. None of the patients exhibited significant tuberous sclerosis symptoms. Among the five patients with PEComa, two patients underwent TH with bilateral adnexectomy. No signs of recurrence were observed in any patient during follow-up. A 46-year-old female was initially diagnosed with uterine fibroids and underwent a single-port laparoscopic myomectomy. An intraoperative frozen-section examination revealed a mesenchymal tumor. TH was not performed at the patient’s family member’s request to preserve the uterus. Recent B-mode ultrasonography at another hospital revealed a 2.3 cm×2.1 cm mass occupying the uterine cavity. However, the patient opted not to undergo further treatment for personal reasons. A 33-year-old female underwent curettage and was diagnosed with PEComa. The patient opted for hysteroscopic lesion resection. Postoperative B-wave ultrasonography did not reveal any specific lesions. However, computed tomography (CT) during follow-up revealed an increase in the size of the subpleural nodules in the right lung to varying degrees. Additionally, multiple new nodular lesions were detected in both lungs, suggesting lung metastasis with possible involvement of the mediastinal and right hilar lymph nodes. Repeat B-ultrasound revealed a uterine mass measuring 5 cm, but the patient declined further treatment. One patient had previously undergone TH due to uterine fibroids. This visit was prompted by the discovery of a pelvic lobulated masses with individual lobes measuring 7 cm and 4 cm. The patient opted for bilateral oophorectomy and resection of the pelvic masses. Postoperative pathology confirmed the presence of PEComa. Whether the tumor was primarily peritoneal or related to the previous uterine fibroids remained unclear. During follow-up, B-mode ultrasound detected a 3.6×1.2×1.7 cm3 hypoechoic lesion in the left pelvic cavity. CT scans also revealed multiple nodules of varying sizes in both lungs, suggesting possible metastasis. The patient declined further treatment due to personal reasons.

One patient diagnosed with a PEComa of UMP underwent TH and bilateral adnexectomy. No signs of recurrence were observed on imaging. Two patients diagnosed with malignant PEComas underwent TH and bilateral adnexectomy without pelvic lymph node dissection. One patient underwent six cycles of epirubicin + ifosfamide chemotherapy. The other patient underwent 28 sessions of radiotherapy, one cycle of ifosfamide + adriamycin chemotherapy, and three cycles of paclitaxel + ifosfamide chemotherapy for possible lymph node metastasis, as evidenced by postoperative CT findings of lymph node enlargement in the right obturator region. No significant signs of recurrence on imaging were observed during follow-up (**Table 4**).

**Table 4:** Diagnosis and treatment of the patients.

Cases	Initial surgery	Initial pathological diagnosis	Final surgery	Final pathological diagnosis	Postoperative chemoradiotherapy	Follow-up Duration (Months)	Survival status	Recent follow-up result
1	single-incision laparoscopic myomectomy	intraoperative frozen section pathological examination: a mesenchymal tumor? an epithelioid smooth muscle tumor or a perivascular epithelioid cell tumor?	single-incision laparoscopic myomectomy (a)	PEComa	no	9	survive	ultrasound revealed a 2.3 × 2.1 cm mass within the uterus at another hospital
2	hysteroscopic lesion resection	PEComa	TH+bilateral adnexectomy (LAP)	PEComa	no	19	survive	no evidence of recurrence
3	bilateral oophorectomy and resection of pelvic mass	intraoperative frozen section pathological examination: hyperplastic fibrovascular tissue with widespread infiltration of inflammatory cells	bilateral oophorectomy and pelvic mass resection	PEComa	no	26	survive	ultrasound revealed a 3.6 x 1.2 x 1.7 cm lesion in the left pelvic cavity. CT revealed multiple nodules of varying sizes in both lungs.
4	curettage	PEComa	TH+bilateral adnexectomy (LAP)	PEComa	no	58	survive	No signs of recurrence were observed in the pelvis. The patient reported undergoing surgery for a pulmonary nodule (details unspecified).
5	curettage	PEComa	hysteroscopic lesion resection	PEComa	no	68	survive	ultrasound revealed a uterine mass measuring 5 cm. CT revealed enlargement of subpleural nodules in the right lung to varying degrees, along with multiple new nodular lesions in both lungs, suggestive of lung metastasis. Mediastinal and right hilar lymph node metastases were also considered possible.
6	hysteroscopic lesion resection	PEComa	TH+bilateral adnexectomy (LAP)	PEComa of UMP	no	9	survive	no evidence of recurrence
7	hysteroscopic lesion resection	intraoperative frozen section pathological examination: the tumor predominantly exhibits epithelioid morphology, with visible mitotic figures (accurately counting the number of mitotic figures in frozen sections is difficult). Focal nuclear atypia is observed. Epithelioid leiomyoma is primarily considered, at least as epithelioid leiomyoma of uncertain malignant potential. Other tumors, such as PEComa and endometrial stromal tumors, also need to be excluded	TH+bilateral adnexectomy (LAP)	malignant PEComa	epirubicin + IFO 6 cycles	19	survive	no evidence of recurrence
8	curettage (b)	neoplastic lesions, PEComa with mitotic count of >1 mitosis/50mm2	TH+bilateral adnexectomy (LAP) in the other hospital	malignant PEComa	radiotherapy 28 times, chemotherapy: IFO+ADM 1 cycle and PTX+IFO 3 cycles (c)	26	survive	no evidence of recurrence

TH total hysterectomy, LAP laparoscope.

a. the patient’s family requested preservation of the uterus during the operation.

b. the patient declined surgery during the initial visit, but underwent total hysterectomy (TH) along with bilateral adnexectomy one year later.

c. postoperative CT revealed lymph node enlargement in the right obturator region, suggesting potential lymph node metastasis.

## 4 Discussion

Current research on the etiology of PEComa primarily focuses on the following (1–3): (1) mutations in tuberous sclerosis complex genes, primarily involving loss of function of the tuberous sclerosis complex 1/tuberous sclerosis complex 2 complex and resulting in increased activation of the mammalian target of rapamycin (mTOR) complex 1 and dysregulation of cell growth signals; (2) rearrangement of the TFE3 gene that may lead to carcinogenic activation by acquiring a strongly expressed promoter; and (3) rearrangement of the RAD51 paralog B gene or other rare gene fusions may play a role. Only one of the eight patients included in this study tested negative for TFE3. The etiology of PEComas remains unclear and requires further research.

PEComa of the female reproductive tract accounts for more than 25% of all PEComa cases, with uterine PEComa being the most prevalent, followed by PEComa affecting the cervix, vagina, broad ligament, round ligament, ovary, and vulva. PEComa can also occur in the pelvis after total or subtotal hysterectomy (4). Recurrence predominantly occurs in the lung, liver, abdomen, vagina, and lymph nodes (5, 6). Uterine PEComa can manifest across a broad age range, spanning from 6 years to 79 years, with a median age of 49 years at onset (5, 6). The clinical manifestations of PEComas lack specificity, and they commonly present as abnormal uterine bleeding, abdominal pain, and compression symptoms. In addition, some patients remain asymptomatic, with imaging revealing only a lower abdominal mass (7). Most tumors are located within the muscle layer. They typically manifest as solitary masses but occasionally occur as multiple masses that are either localized or invasive lesions, with an average diameter of 6.5 cm (range: 0.2–25.0 cm) (5). Some tumors may present with bleeding or necrosis, leading to potential misdiagnosis as uterine fibroids or uterine leiomyosarcoma. The eight patients in this study, aged 33–52 years, exhibited clinical symptoms including abnormal uterine bleeding, vaginal discharge, and pelvic masses. These symptoms are largely consistent with those in previous studies.

PEComa of the female reproductive tract lacks typical imaging manifestations, and it is difficult to make a diagnosis of PEComa before surgery based on imaging features; PEComa is easily misdiagnosed as uterine myoma or sarcoma. Most documented cases of uterine PEComas exhibit heterogeneous echogenicity and prominent central vascularity on ultrasound scans (8). All the patients included in this study underwent ultrasonography, and abundant blood flow signals within and around the masses were common findings, thereby providing valuable diagnostic indications. CT imaging in Patient 2 revealed mild enhancement of the soft tissue, although it was not distinctive. Magnetic resonance imaging provided superior visualization of the internal structure of the lesion, with significant enhancement observed on contrast uptake, offering valuable guidance. In Patient 6, magnetic resonance imaging revealed a nodular lesion measuring 1.5×0.7×1.6 cm in the anterior wall of the lower uterine segment. There was high signal intensity on T2WI, slightly lower than that in the endometrium. Meanwhile, the T1WI signal showed a slightly higher intensity with restricted diffusion. The lesion involved more than half of the anterior wall muscle layer and showed pronounced enhancement on early dynamic scans and mild enhancement on delayed scans. However, prior research has revealed significant disparities in magnetic resonance image characteristics (6). PEComas have the potential to metastasize to the lungs. CT imaging in this study revealed pulmonary nodules in multiple patients, including those with “benign” PEComa. Surgical intervention was even required in one patient, emphasizing the necessity of vigilance. However, this study did not acquire pathological evidence of pulmonary nodules, indicating the need for further observations in future studies.

PEComas are composed of epithelioid and spindle cells. A crucial morphological characteristic for diagnosing PEComas is the presence of delicate vascular channels surrounding tumor cells and nests, which are atypical for smooth muscle tumors (9). Microscopically, PEComa tumors exhibit rich vascularity with thin vessel walls. Tumor cells are arranged in lamellar or nest-like patterns around blood vessels, displaying polymorphic shapes such as polygonal, oval, circular, and spindle shapes. The cells have well-defined borders; abundant and delicate cytoplasm with granular, eosinophilic, oval, or round nuclei; visible nucleoli; and heterogeneous mitotic images. Other characteristics of smooth muscle tumors include perinuclear vacuoles and a diffuse eosinophilic cytoplasm. Immunohistochemically, the perivascular epithelioid cells express melanocyte and myogenic differentiation markers (10). The important melanin markers include human melanoma black 45 (HMB45), melan-A, melanocyte-inducing transcription factor, and cathepsin K. In uterine PEComa, PNL2 exhibits high specificity and sensitivity (11), however, there are variations in the types and expression levels of melanocyte markers reported across studies (12). Common myogenic markers include smooth muscle actin, desmin, and heavy calmodulin-binding proteins (caldesmon). Furthermore, malignant PEComas frequently demonstrate TP53, RB1, and ATRX inactivation (13). Other tumor types such as leiomyosarcoma may present with PEComa-like characteristics, necessitating the combination of various types of data, including genomic profiles, for diagnosis (14). Immunohistochemically (**Table 3**), all tumors were positive for HMB45 and/or melan-A (**Figure 1**). Additionally, the tumors were positive for at least one myogenic marker, with the positivity rate being the highest for desmin (5/8), followed by caldesmon (3/8), smooth muscle actin (3/8), and calponin (3/8). This finding is slightly different from those reported in previous studies (15). Furthermore, positivity rates for VIM (4/8) and cyclin D1 (3/8) were notably high. One patient with malignant PEComa tested positive for TFE3. Most patients were also positive for the estrogen receptor (5/8) and progesterone receptor (7/8) (**Figure 1**). Consideration of uterine PEComa as a hormone-dependent disease warrants further investigation. Studies have suggested a cross-interaction between the mTOR pathway and estrogen receptor signaling, where estrogen E2 binding to the receptor activates Akt/mTOR, thereby mediating PEComa resistance to mTOR inhibitors. Sanfilippo et al. retrospectively evaluated seven patients treated with a combination of an mTOR inhibitor (sirolimus) and an anti-estrogen therapy; the results showed significant clinical efficacy, with a disease control rate of 86% (16). Additionally, our study found that malignant tumors and PEComas of UMP exhibited high Ki67 values. This suggests a correlation between the Ki67 value and PEComa risk, as well as the degree of malignancy, warranting further investigation. The pathological differential diagnosis of PEComas of the uterus and pelvic cavity includes all mesenchymal neoplasms showing spindle and/or epithelioid cell features, such as smooth muscle tumors and endometrial stromal sarcomas. Compared with smooth muscle tumors, PEComas have key features of eosinophilic to clear cytoplasm, round to oval nuclei, a prominent capillary network, and strong immunoreactivity for melan-A, HMB45, melanocyte-inducing transcription factor, and cathepsin K. Furthermore, PEComas commonly stain negatively for epithelial membrane antigen and cytokeratin, unlike epithelioid smooth muscle tumors. Endometrial stromal sarcomas can resemble PEComas, particularly the epithelioid variants, because both tumors may exhibit similar infiltrative patterns within the myometrium. However, endometrial stromal sarcomas exhibit morphological features, such as endometrial involvement and stromal differentiation. Additionally, immunohistochemical markers such as CD10+, HMB45-, melan-A-, and melanocyte-inducing transcription factor help to differentiate them from PEComas (6).

In 2005, Folpe et al. proposed the following criteria to assess the risk level of PEComa (17), where two or more of the following criteria were required for malignant diagnosis: tumor diameter >5.0 cm, infiltration, high nuclear grade, mitotic count of >1 mitosis/50 mm^2^, necrosis, and vascular invasion. A benign PEComa was considered if the following characteristics were found: tumor diameter <5 cm, non-infiltrative, non-high nuclear grade, mitotic count ≤1 mitosis/50 mm^2^, no necrosis, and no vascular invasion. If only nuclear pleomorphism/multinucleated giant cells were present or if the maximum diameter of the tumor was >5 cm, the classification was a PEComa of UMP. Thereafter, the revised risk assessment system (15) proposed five clinicopathological features to predict the biological behavior of PEComa: tumor diameter ≥5 cm, high nuclear grade, mitotic count >1 mitosis/50 mm^2^, necrosis, and vascular invasion. When a tumor exhibits three or more features, it is classified as malignant, whereas a tumor with two or fewer features is considered as a PEComa of UMP. As recommended, the use of the term “benign” should be minimized. These criteria were adopted in the 2020 World Health Organization classification of tumors of the female reproductive system (5). The current study shows the typical pathological manifestations of PEComa (**Figure 2**), PEComa of UMP (**Figure 3**), and malignant PEComa (**Figure 4**), presenting the distinctive characteristics and differences among the three types. Pathologists should carefully differentiate, as the pathologic diagnosis will influence the treatment plan.

PEComas of the female reproductive tract pose challenges during tumor biological behavior assessment. The primary treatment approach for PEComa of the female reproductive tract involves complete resection with negative incisional margins. TH is currently the primary treatment modality for uterine PEComas. In theory, PEComas primarily metastasize hematologically, suggesting the limited significance of lymphadenectomy (18). Currently, lymphadenectomy is not a routine component of surgical treatment for PEComa, but it is recommended for patients with lymph node metastasis (19). Given the limited number of PEComa cases, further studies are warranted to establish a basis for treatment decision making. In addition, chemoradiotherapy should be considered when managing malignant PEComas (20). The staging of malignant PEComas should follow that of uterine sarcomas. In patients with inoperable or residual lesions, single or combination chemotherapy with dacarbazine, isocyclophosphamide, doxorubicin, vincristine, or other antitumor drugs may be considered (6, 19). Owing to the limited number of clinical cases and lack of long-term follow-up data, the efficacy of chemotherapy regimens remains unclear.

Recurrence and metastasis can manifest locally or distantly, affecting organs such as the liver, lungs, intestines, bones, and lymph nodes (21), and may emerge months or even decades after surgery (22). The management of recurrent and/or distant metastatic PEComa remains uncertain, but surgery is the optimal treatment modality for isolated recurrent lesions in patients in good physical condition. Chemotherapy may be attempted, and targeted therapies, such as the mTOR inhibitors sirolimus and everolimus (7, 23), also appear to be promising (24). Immunotherapy using programmed cell death 1 inhibitors and vascular endothelial growth factor receptor inhibitors may be used as second-line or subsequent-line treatments, particularly for patients experiencing resistance to mTOR inhibitors and disease progression (25, 26). In young women requiring fertility preservation, meticulous consideration should be given to simple tumor resection if the tumor is small or benign or exhibits UMP. However, in our study, two patients experienced recurrence of uterine masses shortly after simple tumor resection, whereas two patients diagnosed with malignant PEComa who underwent TH and bilateral adnexectomy followed by chemotherapy, with or without radiotherapy, exhibited favorable prognoses without recurrence. This indicates that preservation of the uterus in patients with PEComa should involve strict patient selection and detailed counseling regarding the risks of recurrence and metastasis. Moreover, with appropriate treatment, satisfactory outcomes can be achieved for malignant PEComas.

## 5 Conclusion

PEComa of the female reproductive tract is a rare tumor that often presents with atypical signs and symptoms; thus, it is frequently misdiagnosed as other uterine tumors preoperatively. Diagnosis primarily relies on pathology, which poses challenges for early identification. The low incidence rates and limited availability of clinical data hinder a comprehensive understanding of the risk and invasiveness of uterine or pelvic PEComa. Although the current criteria are generally effective in assessing tumor invasiveness, emphasizing the significance of complete lesion resection is crucial. Inadequate treatment significantly increases the risks of recurrence and metastasis. Additionally, the prevalence of pulmonary metastases may have been underestimated. Refining risk stratification to prevent the overtreatment of low-grade malignancies and overlooking highly aggressive tumors is a pertinent research avenue.

## Data Availability

The original contributions presented in the study are included in the article/supplementary material. Further inquiries can be directed to the corresponding author.
